# Associations between food insecurity and Sleep Duration, Quality, and Disturbance among older adults from six low‐ and middle‐income countries

**DOI:** 10.1016/j.jnha.2023.100018

**Published:** 2024-01-01

**Authors:** Pishva Arzhang, Narges Sadeghi, Fatemeh Ahmadi Harchegani, Mahsa Rezaei, Moslem Ghaderi, Mir Saeed Yekaninejad, Cindy W. Leung, Leila Azadbakht

**Affiliations:** a Qods Hospital, Kermanshah University of Medical Sciences, Kermanshah, Iran; b Nutrition and Metabolic Diseases Research Center, Ahvaz Jundishapur University of Medical Sciences, Ahvaz, Iran; c Department of Epidemiology and Biostatistics, School of Public Health, Tehran University of Medical Sciences, Tehran, Iran; d Department of Clinical Nutrition, School of Nutritional Sciences and Dietetics, Tehran University of Medical Sciences, Tehran, Iran; e School of Nursing and Midwifery, Iran University of Medical Sciences, Iran; f Department of Epidemiology and Biostatistics, School of Public Health, Tehran University of Medical Sciences, Tehran, Iran; g Department of Nutrition, Harvard T.H. Chan School of Public Health, Boston, MA, USA; h Department of Community Nutrition, School of Nutritional Sciences and Dietetics, Tehran University of Medical Sciences, PO Box: 1416643931, Tehran, Iran

**Keywords:** Food insecurity, Sleep duration, Sleep quality, Sleep disturbance, Low‐ and middle‐income countries

## Abstract

**Objectives:**

Although food insecurity has been associated with poor sleep outcomes in young and middle-aged adults, few studies have examined this relationship in older adults. This study aimed to examine the relationship between food insecurity and sleep duration, quality, and disturbance among older adults in six low-income countries (LMICs).

**Design and Setting:**

We analyzed nationally representative cross-sectional data from 33,460 adults (≥50 years) from the Study on global AGEing and adult health (SAGE).

**Measurements:**

Food insecurity (FI) was assessed using two questions regarding the frequency of eating less and hunger caused by a lack of food. Sleep outcomes included self-reported sleep duration, sleep quality, and sleep disturbances (difficulty falling asleep, frequent sleep interruptions, and early awakening) assessed based on self-reports over two nights. Multivariable logistic regression was used to assess country-specific relationships between food insecurity and sleep outcomes, and random-effects models were used to estimate pooled associations.

**Results:**

The prevalence of FI among older adults in the overall population was 16.2%. In pooled analyses, FI was significantly associated with long sleep duration ≥ 9 h (OR=1.58, 95% CI: 1.30 to 1.93; P=0.001). There were also significant pooled associations between FI and poor sleep quality (OR=1.34, 95% CI: 1.14 to 1.56; P < 0.001) and sleep disturbances (OR=1.44, 95% CI: 1.08 to 1.91; P = 0.014).

**Conclusions:**

In conclusion, the current study found that FI is adversely associated with sleep duration, quality and disturbances in older adults, with some heterogeneity by country. The findings suggest food policies and intervention programs are needed for vulnerable households.

## 1 Introduction

Food insecurity (FI) is a state in which people do not have sufficient access to safe and nutritious foods to maintain optimal growth and development and live in an active, healthy life. FI emerges when there are not enough foods available or people lack enough money and resources to provide food for their household [1]. In 2021, moderate or severe FI affected almost 2.3 billion people worldwide [2]. FI poses a greater health risk and is more prevalent in older adults [3]. More than 2.9 million households with an adult ≥65 years were food insecure in 2018 [4]. Previous literature shows that food insecurity among older adults is associated with several adverse health consequences [5]. FI and poor nutritional status in the elderly is not only linked to chronic physical concerns such as diabetes, hypertension, and higher cardiovascular risk, but also to depression and poor mental health [6]. Poor mental health can exacerbate physical health conditions, which can increase health care costs and the need for social support; therefore, food insecurity is an important social determinant of health for older adults [7].

One of the key behavioral factors related to mental health is sleep [8]. Traditionally, sleep disturbances were considered a consequence of mental disorders like depression. However, evidence now suggests a reverse association between sleep and mental health, such that sleeping disturbances can impair cognitive function, affect emotion regulation, and precede the onset of psychiatric disorders probably [[8], [9], [10]]. Poor sleep quality has been adversely associated with physical and mental health and quality of life [11], and even all-cause mortality in older adults [12]. According to previous studies, the prevalence of chronic sleeping problems in older adults ranges from 40 to 70% and half of the cases are undiagnosed [13]. Therefore, it is crucial to identify the factors influencing sleep duration and quality in older adults. Food insecurity is a potential factor linked to poor sleep quality [[14], [15], [16]]. The mechanisms involved between FI and sleeping disorders are yet to be elucidated; however, FI seems to exert its detrimental effects on sleep quality by provoking other risk factors of poor sleep, such as stress, psychological distress, and depression [14,15].

Although research efforts have widely aimed at understanding the health implications of food insecurity among children and younger-aged adults, little is known about the relationship between food insecurity and sleep quantity and quality among older adults [6,14]. Considering the fact that food insecurity and sleep disturbances may be more concentrated in areas of lower socioeconomic status, it is important to investigate the relationship between FI and sleep disturbances particularly in low- and middle-incomes countries (LMICs) where economic growth is lagging and FI is more concentrated [14,17]. The aim of the present study is to examine the association between food insecurity and sleep outcomes in a sample of older adults from six LMICs.

## 2 Methods

### 2.1 Study design

**Fig. 1:**
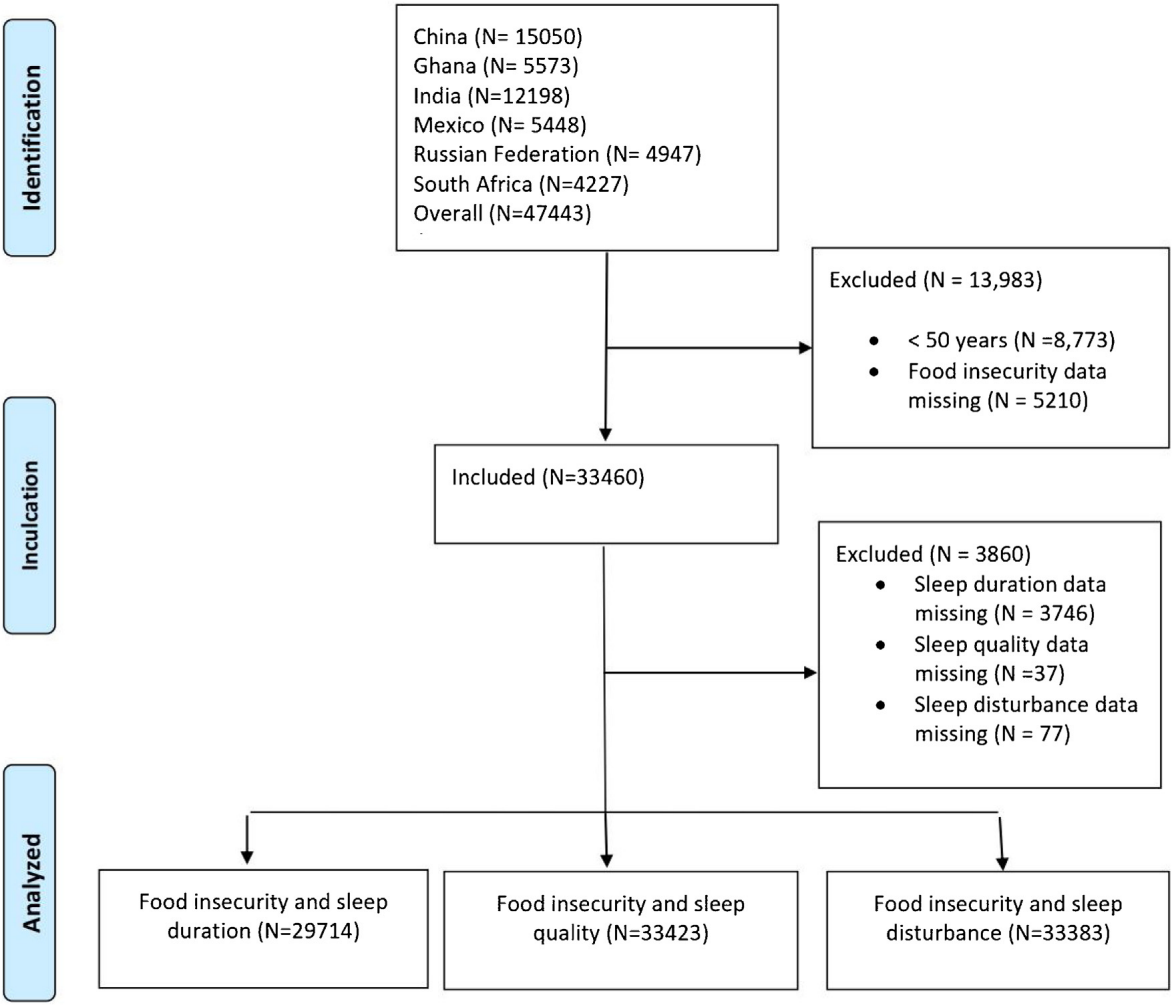
Participant Flow Chart.

The data used in the present study is obtained from the WHO’s longitudinal Study on global AGEing and adult health (SAGE) wave 1 which consists of cross-sectional surveys from large nationally representative samples of adults 50 years and older from six LMICs: Ghana, China, Russian Federation, India, South Africa and Mexico, between 2007 and 2010. The WHO Health Survey used a multistage, stratified, random-clustered sampling design via weight sampling reported by the United Nations Statistical Division to collect data generalizable to population structures. Adults 50 years and older from the six LMICs who provided written informed consent before the study were eligible to participate in this research. Interviewers administered standard questionnaires in the participant’s native language. Participants with missing data on food insecurity (n = 8,861) were excluded. The overall analytical sample consisted of 33,460 adults aged 50 years and older in China (n = 12,936), Ghana (n = 4,261), India (n = 6,555), Mexico (n = 2,209), Russian Federation (n = 3,874), South Africa (n = 3,625). See participant flow chart in Fig. 1. The SAGE protocol was approved by WHO Ethical Review Committee and local ethics research review boards [18].

### 2.2 Food insecurity (Exposure)

To assess the food insecurity status, two questions adapted from the US Household Food Security Survey Module were used. The questions assessed the frequency of eating less than enough due to the lack of sufficient food and the frequency of being hungry due to not affording enough food in the last 12 months. The response options of both questions ranged from 1 (every month) to 5 (never). At the beginning, we classified food security status as: [1] Food secure: responded 5 to both questions [2]; Moderately food insecure: responded 2–4 to either of the questions; and [3] Severely food insecure: responded 1 to at least one question or 2–3 to either of the questions [19,20]. However, in the present study, we merged severe and moderate food insecure groups as “food insecure” group and analyzed food insecurity status as a dichotomous variable (food secure and food insecure) [[21], [22], [23]].

### 2.3 Sleep variables (Outcomes)

Sleep duration, sleep quality and sleep disturbances were evaluated for all participants. To assess sleep duration, participants reported the duration of their sleep for each of the last two nights prior to the time of interview excluding the daytime naps. The average sleep length of both nights was calculated and categorized into three levels of sleep duration: short sleep (≤ 6 h), intermediate sleep (>6−9 hours), and long sleep (>9 h). To assess the sleep quality, participants were asked to rate their sleep quality on a Likert scale ranging from 1 (very good) to 5 (very poor). Sleep quality scores were reverse coded for analysis such that higher scores reflected better sleep quality. Sleep quality was then dichotomized into poor sleep quality [[1], [2], [3]] or high sleep quality [4,5,10]. To assess sleep disturbances, participants responded to a single question assessing the presence of any sleep problems over the last 30 days, including difficulty falling asleep, frequent sleep interruptions, or waking up too early. Responses ranged from none [1] to extreme [5]. Sleep disturbance was categorized as anyone who chose severe [4] or extreme [5,24].

### 2.4 Control variables

The control variables of the study included gender, age, race, wealth quintiles based on income, smoking status, alcohol use in the last 30 days, BMI and numerous of physical conditions (Asthma, Chronic lung disease, Diabetes, Hypertension and Hearing problem) selected based on the literature review [10,14,20].

### 2.5 Statistical analysis

Statistical analyses were performed using STATA software version 14.2. To investigate the association between FI and sleep outcomes, multivariable logistic regression analysis was performed stratified by country, adjusting for all control variables. Between-country heterogeneity in the relationship between FI and sleep outcomes was measured by Higgins’s I^2^ according to estimates from each country. Odds ratios (ORs) and 95% confidence intervals (CIs) are shown for the findings. To produce the pooled estimates, random-effect meta-analysis was used. All results were considered significant with P-value ≤ 0.05.

## 3 Results

### 3.1 Sample characteristics

The prevalence of food insecurity in the overall population was 16.2%. In total, 61.9% of the overall population was under 65 years old, and 54% were females. Moreover, 50.1% lived in urban areas, 10.8% were underweight, 27.4% were smoking and 19.5 % were current alcohol consumers. More information about the sample characteristics of each country is provided in Table 1.

**Table 1:** Socio-demographic characteristics among older adults (≥50 years) in six low- and middle-incomes countries.

Variables^a^	China (N = 12936)	Ghana (N = 4261)	India (N = 6555)	Mexico (N = 2209)	Russian Federation (N = 3874)	South Africa (N = 3625)	Overall (N = 33460)
Food security							
Secure	12773 (98.7)	2316 (54.4)	5406 (82.5)	1590 (72.0)	3353 (86.6)	2587 (71.4)	28025 (83.8)
Insecure	163 (1.3)	1945 (45.6)	1149 (17.5)	619 (28.0)	521 (13.4)	1038 (28.6)	5435 (16.2)
Age							
Age ≤65	8136 (62.9)	2507 (58.8)	4662 (71.1)	1013 (45.9)	2042 (52.7)	2356 (65.0)	20716 (61.9)
Age >65	4800 (37.1)	1754 (41.2)	1893 (28.9)	1196 (54.1)	1832 (47.3)	1269 (35.0)	12744 (38.1)
Gender							
Male	6066 (46.9)	2224 (52.2)	3301 (50.4)	872 (39.5)	1372 (35.4)	1533 (42.3)	15368 (45.9)
Female	6870 (53.1)	2037 (47.8)	3254 (49.6)	1332 (60.3)	2502 (64.6)	2090 (57.7)	18085 (54.0)
Area							
Urban	6355 (49.1)	1742 (40.9)	1676 (25.6)	1602 (72.5)	2951 (76.2)	2423 (66.8)	16749 (50.1)
Rural	6581 (50.9)	2519 (59.1)	4879 (74.4)	607 (27.5)	923 (23.8)	1197 (33.0)	16706 (49.9)
BMI							
Under weight	568 (4.4)	635 (14.9)	2239 (34.2)	19 (0.9)	29 (0.7)	138 (3.8)	3628 (10.8)
Normal Weight	7663 (59.2)	2330 (54.7)	3203 (48.9)	506 (22.9)	801 (20.7)	890 (24.6)	15393 (46.0)
Overweight	3436 (26.6)	781 (18.3)	722 (11.0)	813 (36.8)	1445 (37.3)	987 (27.2)	8184 (24.5)
Obese	694 (5.4)	400 (9.4)	190 (2.9)	647 (29.3)	1198 (30.9)	1469 (40.5)	4598 (13.7)
Smoking							
Don't Smoke	9415 (72.8)	3717 (87.2)	3453 (52.7)	1805 (81.7)	3180 (82.1)	2657 (73.3)	24227 (72.4)
Smoke	3480 (26.9)	534 (12.5)	3102 (47.3)	404 (18.3)	689 (17.8)	945 (26.1)	9154 (27.4)
Alcohol							
Yes	2629 (20.3)	1323 (31.0)	485 (7.4)	298 (13.5)	1225 (31.6)	549 (15.1)	6509 (19.5)
No	10250 (79.2)	2919 (68.5)	6070 (92.6)	1911 (86.5)	2648 (68.4)	3044 (84.0)	26842 (80.2)

BMI: body mass index.

a Data are Number (percent).

### 3.2 Associations between food insecurity and sleep duration

The country-specific associations between FI and sleep duration are shown in Table 2. In China, food insecurity was positively associated with both short sleep duration (OR = 1.86, 95% CI: 1.26–2.76; P = 0.002) and long sleep duration (China: OR = 2.22, 95% CI: 1.41–3.49; P = 0.001). In Ghana, food insecurity was inversely associated short sleep duration (OR = 0.63, 95% CI: 0.51 to 0.78; P < 0.001) and positively associated with long sleep duration (OR = 1.43, 95% CI: 1.15–1.78; P < 0.001). In India, food insecurity was related to long sleep duration (OR = 1.69, 95% CI: 1.25–2.28; P = 0.001), but not associated with short sleep duration. In contrast, in the Russian Federation food insecurity was associated with short sleep duration (OR = 1.44, 95% CI: 1.09–1.90; P = 0.008), but not associated with long sleep duration. Lastly, in South Africa, food insecurity was associated with long sleep duration (OR = 1.81, 95% CI: 1.53–2.16; P < 0.001), but not short sleep duration. Data for sleep duration in Mexico were not reported in SAGE documents.

**Table 2:** Association between food insecurity and sleep duration among older (≥50 years) adults from six low‐ and middle‐income countries.

	Sleep duration						
	≤6 h			7−8 h	≥9 h		
	OR	95% CI	P-value^a^	Ref	OR	95% CI	P-value
China							
Adjusted^b^	1.86	1.26 to 2.76	0.002	Ref	2.22	1.41 to 3.49	0.001
Crude	1.93	1.32 to 2.82	0.001	Ref	2.35	1.54 to 3.58	<0.001
Ghana							
Adjusted^b^	0.63	0.51 to 0.78	<0.001	Ref	1.43	1.15 to 1.78	0.001
Crude	0.60	0.49 to 0.73	<0.001	Ref	1.43	1.18 to 1.75	<0.001
India							
Adjusted^b^	1.00	0.84 to 1.18	0.964	Ref	1.69	1.25 to 2.28	0.001
Crude	1.00	0.85 to 1.16	0.988	Ref	2.04	1.57 to 2.66	<0.001
Russian Federation							
Adjusted^b^	1.44	1.09 to 1.90	0.008	Ref	1.07	0.76 to 1.52	0.689
Crude	1.52	1.18 to 1.95	0.001	Ref	1.23	0.90 to 1.70	0.184
South Africa							
Adjusted^b^	0.87	0.62 to 1.22	0.421	Ref	1.81	1.53 to 2.16	<0.001
Crude	0.80	0.59 to 1.08	0.154	Ref	2.05	1.75 to 2.4	<0.001

a P values were calculated using multivariable logistic regression.

b Adjusted for gender, age, race, wealth quintiles based on income, smoking, alcohol use, BMI.

**Fig. 2:**
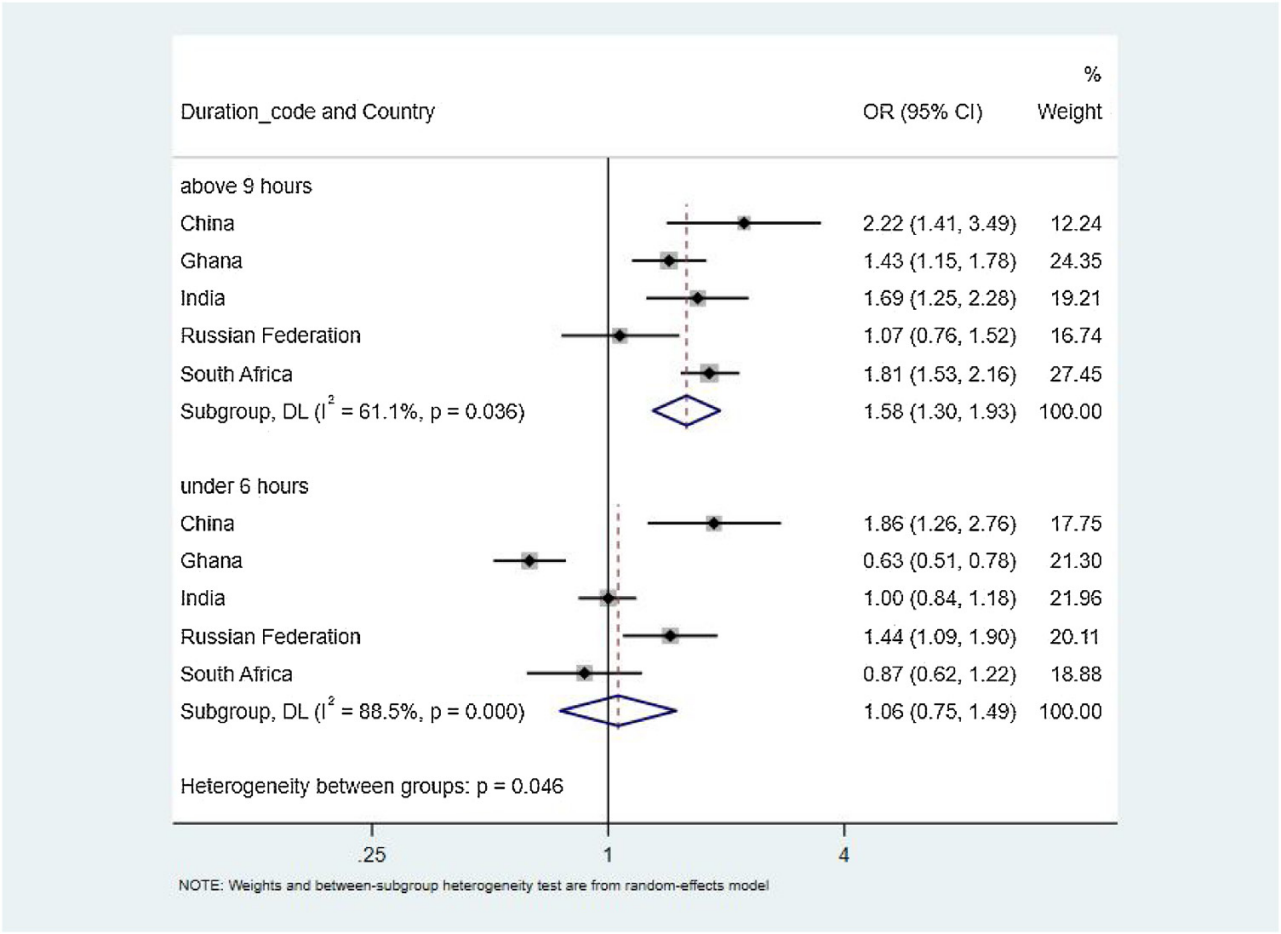
Country-Wise Association Food Insecurity with sleep duration among older adults estimated by multivariable logistic regression.

The pooled associations between food insecurity and sleep duration are reported in Fig. 2. For the outcome of short sleep duration, there was a high level of between-country heterogeneity (I^2^ = 88.5%) and no significant overall association between FI and short sleep (OR = 1.06, 95% CI: 0.75–1.49; P = 0.735). For long sleep duration, there was a high level of between-country heterogeneity (I^2^ = 61.1%) and a significant overall association between FI and long sleep (OR = 1.58, 95% CI: 1.30–1.93; P < 0.001).

### 3.3 Associations between food insecurity and sleep quality

Table 3 present country-specific associations between food insecurity and sleep quality and sleep disturbances. For the outcome of poor sleep quality, significant positive associations were observed with food insecurity in China (OR = 1.41, 95% CI: 1.01–1.95; P = 0.040), Ghana (OR = 1.32, 95% CI: 1.15–1.52; P < 0.001), Mexico (OR = 1.39, 95% CI: 1.10–1.75; P = 0.005), Russian Federation (OR = 1.50, 95% CI: 1.13–1.85; P = 0.003), and South Africa (OR = 1.57, 95% CI: 1.32–1.87; P < 0.001). No significant associations were observed in India.

**Table 3:** Association between food insecurity with sleep quality and Sleep disturbance among older adults (≥50 years) from six low‐ and middle‐income countries.

	Sleep quality			Sleep Disturbance		
	OR	95% CI	P-value^a^	OR	95% CI	P-value
China						
Adjusted^b^	1.41	1.01 to 1.95	0.040	2.03	0.98 to 4.24	0.057
Crude	1.49	1.09 to 2.04	0.012	2.25	1.14 to 4.45	0.019
Ghana						
Adjusted^b^	1.32	1.15 to 1.52	<0.001	0.80	0.62 to 1.03	0.088
Crude	1.39	1.22 to 1.58	<0.001	0.83	0.65 to 1.05	0.123
India						
Adjusted^b^	1.01	0.89 to 1.17	0.853	1.33	1.09 to 1.61	0.004
Crude	1.26	1.10 to 1.43	<0.001	1.72	1.45 to 2.03	<0.001
Mexico						
Adjusted^b^	1.39	1.10 to 1.75	0.005	1.51	1.01 to 2.25	0.044
Crude	1.44	1.16 to 1.77	0.001	1.32	0.93 to 1.87	0.117
Russian Federation						
Adjusted^b^	1.50	1.13 to 1.85	0.003	1.69	1.28 to 2.23	<0.001
Crude	1.77	1.43 to 2.20	<0.001	2.03	1.60 to 2.56	<0.001
South Africa						
Adjusted^b^	1.57	1.32 to 1.87	<0.001	1.94	1.49 to 2.53	<0.001
Crude	1.58	1.35 to 1.85	<0.001	2.14	1.69 to 2.70	<0.001

a P values were calculated using multivariable logistic regression.

b Adjusted for gender, age, race, wealth quintiles based on income, smoking, alcohol use, BMI.

**Fig. 3:**
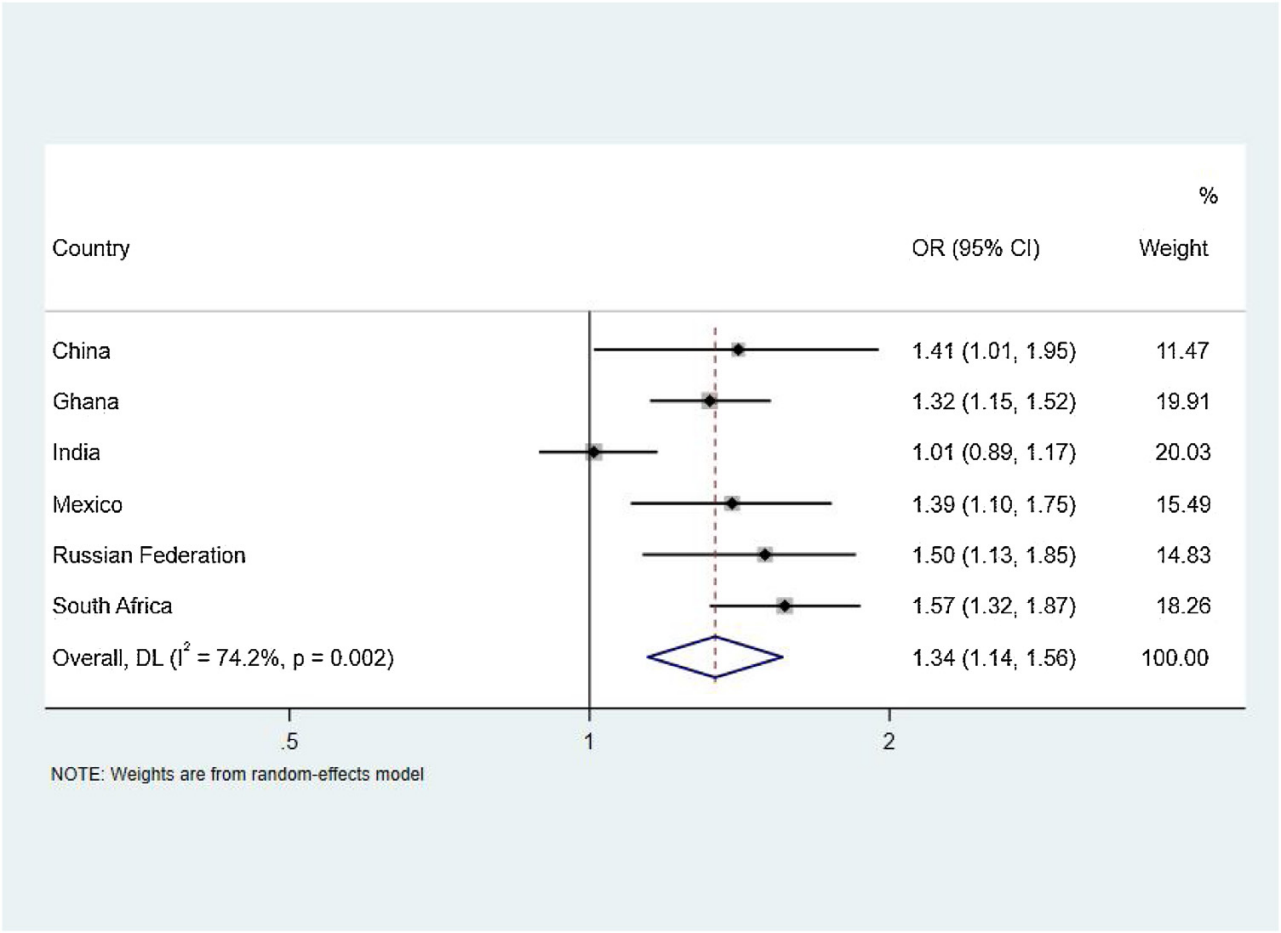
Country-Wise Association Food Insecurity with poor sleep quality among older adults estimated by multivariable logistic regression.

Fig. 3 demonstrates the pooled association between FI and poor sleep quality. Despite some evidence of high between-country heterogeneity (I^2^ = 74.2%), there was a significant overall association between FI and poor sleep quality (OR = 1.34, 95% CI: 1.14–1.56; P < 0.001).

### 3.4 Associations between food insecurity and sleep disturbance

As shown in Table 3, food insecurity was positively associated with sleep disturbances in India (OR = 1.33; 95% CI: 1.09–1.61; P = 0.004), Mexico (OR = 1.51; 95% CI: 1.01–2.25; P = 0.044), Russia (OR = 1.69; 95% CI: 1.28–2.23; P < 0.001) and South Africa (OR = 1.94; 95% CI: 1.49–2.53; P < 0.001). No significant associations were observed in China and Ghana.

**Fig. 4:**
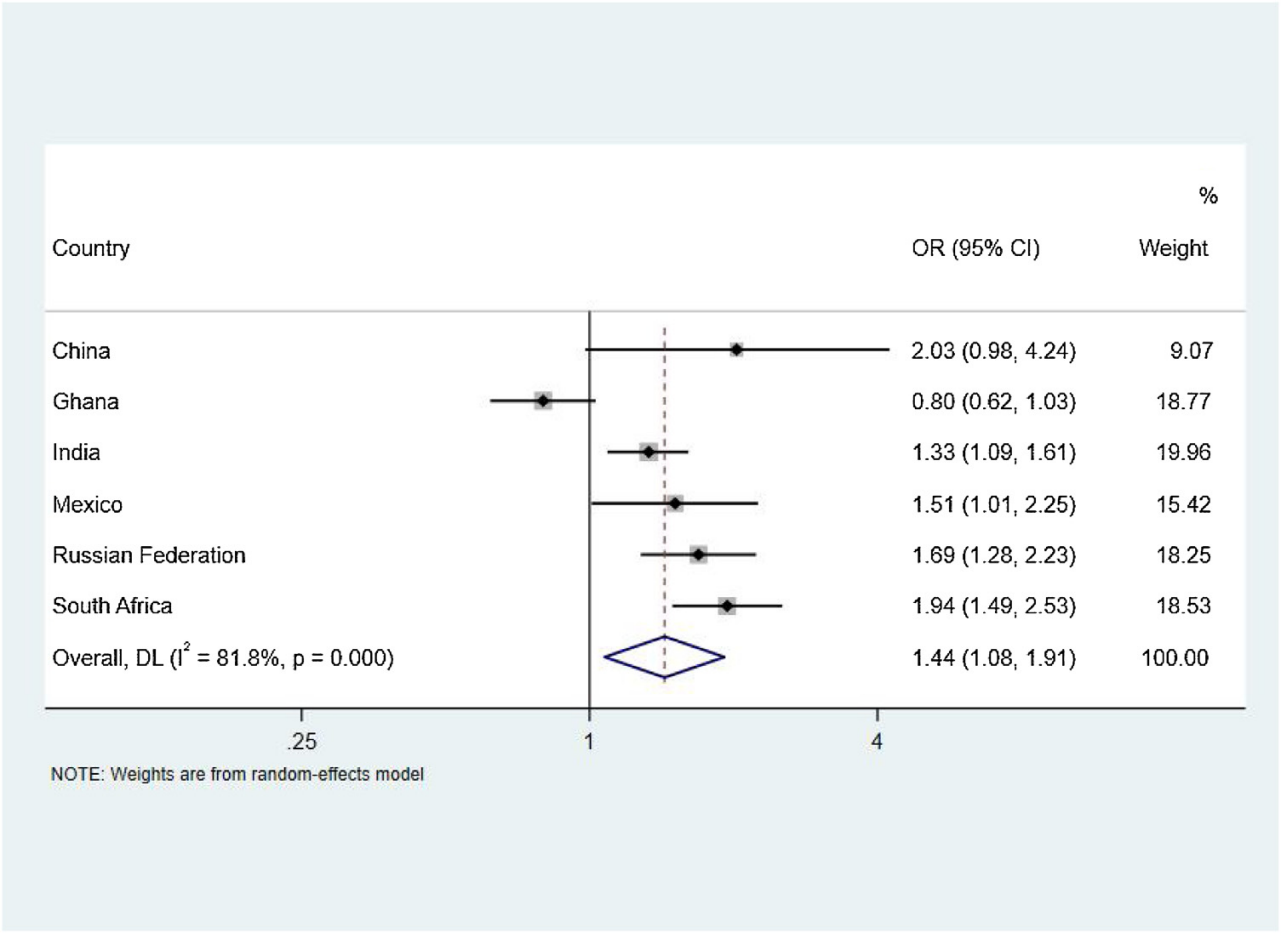
Country-Wise Association Food Insecurity with sleep disturbance among older adults estimated by multivariable logistic regression.

The pooled association between FI and sleep disturbance is reported in Fig. 4. There was a high level of between-country heterogeneity (I^2^ = 81.8%) and significant overall association between FI and sleep disturbance (OR = 1.44, 95% CI: 1.08–1.91; P = 0.014).

## 4 Discussion

Results of the present study showed a positive and significant associations between FI and longer sleep duration in all countries, but Russia. Moreover, there was also a positive and significant association between FI and poor sleep quality in all countries, except for India. Ultimately, FI was positive and significant associated with sleep disturbance in all countries except for China and Ghana.

Our findings build on several previous studies investigating the relationship between FI and sleep health. A study [25] found that adult women with very low food security reported significantly shorter sleep durations than adult women with full food security. Furthermore, Troxel et al. [26] studied 785 adults living in two low-income neighborhoods in the US and found that FI was associated with poorer sleep efficiency and shorter sleep duration in adults. Another study [27] found that the prevalence of self-reported insufficient sleep duration (<7 h) was greater among adults those that reported greater levels of FI. Jordan et al. [28] reported a significant association between severe household FI and getting less than the recommended amount of sleep (≤6 h). Data among 1201 participants from the AgeHeaPsyWel-HeaSeeB survey in Ghana showed older adults with moderate and severe FI had fewer sleep hours than those who were food secure [29]. While most prior studies have consistently shown that food insecurity is associated with shorter sleep duration, as we also found in China and Russian Federation, our study more consistently showed that food insecurity was associated with longer sleep duration among older adults. Still, it is noteworthy that out of the 5 studies above, 4 were conducted among adults of all ages as participants. Therefore, this inconsistency could be due to lack of data on relationship between FI and sleep duration in older adults. As far as we know, the AgeHeaPsyWel-HeaSeeB study was the only other study that focused on older adult participants; yet, the inconsistent results in this study could be related to lower sample size.

A wide range of factors have been correlated with long sleep duration, including demographic, lifestyle, socioeconomic, medical, psychological, dietary quality and sleep-related variables. In the longstanding US-based Nurses’ Health Study [30], women with past depressive symptoms were 1.7 times as likely to have long sleep and women with current depressive symptoms were 2.9 times as likely to have long sleep, compared to those without depressive symptoms. Individuals who live alone more likely to be a long sleeper. Additionally, low socioeconomic statuses, such as lack of employment, low household income, or low perceived social status, were strongly associated with longer sleep hours [30,31]. On the other hand, prior research has shown that poor dietary intake is associated with food insecurity [32], and dietary quality is associated with both short and long sleep duration [33]. While there is substantially less evidence in the literature showing that long sleep duration is associated with lower diet quality, a few other research have found association between higher consumption of unhealthy food [32] and less intake of vegetables and fruits [34,35] with longer sleep duration.

We found a significant association between FI and poor sleep quality. Aligned with our findings, Jordan et al. [28] found that the increasing severity of FI was significantly associated with poor sleep quality among Mexican adults. Furthermore, Troxel et al. [26] studied 785 adults living in two low-income neighborhoods in the US and found that FI was associated with poorer sleep efficiency, shorter sleep duration, and poorer subjective sleep quality as measured by actigraphy. In a multi-campus study, Hagedorn et al. [36] found that students who were food insecure had higher adjusted odds of poor sleep quality. As well as, children with FI had 2.27-fold higher odds of poor sleep quality, according to Na et al. [37] study. Nagata et al. [38] determine the association between FI and sleep outcomes among young adults in the US and found that FI associated with poorer sleep outcomes including trouble falling and staying asleep. Moderate and severe levels of food insecurity were positively and significantly associated with poor sleep quality, according to Gyasi et al. [29] study. El Zein et al. [39] reported that in comparison to food-secure students, first-year college students with food insecurity were two times more likely to have poor sleep quality. The majority of prior studies on FI and sleep quality were done among adolescents and younger-aged adults. This study stands out because it is one of few studies among older adults.

The results of our study show that there is a positive association between FI and sleep disturbances. Similarly, Wang [24] analyzed self-reported data from adolescents in 68 countries and found that severe FI was significantly associated with a higher risk of sleep disturbance in 48 of the 68 countries after adjusting for covariates. Also, Isaura et al. [40] found that food-insecure individuals are more likely to experience sleep disturbances.

Some biological hypotheses may explain the association between FI and poorer sleep outcomes. A scarcity of essential food or pecuniary resources can cause starvation and a lack of energy, potentially leading to nutritional deficiencies [29]. Individuals experiencing food insecurity tend to eat less nutritious foods, especially those that contain more carbohydrates, fats, and refined sugars and fewer minerals, proteins, vitamins, and micronutrients [20]. There is a possibility that malnutrition (mostly protein-energy malnutrition) can lead to low tryptophan levels, which may affect the synthesis of melatonin, a neuro-hormonal acid that can regulate circadian rhythm and promotes falling asleep and maintaining it [41]. Sleep quality might be affected by eating an unhealthy or disordered diet, which could decrease the energy required for sleep initiation [42]. Previous research has shown unhealthy eating habits are strongly associated with poor sleep quality [33,[42], [43], [44]]. People with low education levels are more likely to hold manual labor jobs and receive minimum wages, which could make it difficult for them to maintain adequate nutrition through healthy meals. It is also possible that low education may lead to fewer opportunities for nutrition education explaining how to maintain a well-balanced, nutritious diet [45,46].

Food-insecure adults may have diets deficient in nutrients, such as vitamin B-12 or folic acid, which can affect mood and immune function, resulting in sleep disturbances [47]. FI is related to depressive symptoms, and having depressive symptoms increases the odds of experiencing sleep disturbance [48]. There is a 2.57 times higher risk of anxiety and a 2.53 times higher risk of depression among food-insecure individuals who lack concentration, are sad, anxious, and do not engage in intellectually stimulating activities [49]. In a study which was done on SAGE (the same data as current study) the results showed that stress and depression play mediatory role between FI and insomnia related symptoms [21]. Furthermore, a person experiencing depression may not be as motivated, energetic, or organized to shop, cook, or make healthy food choices [50]. Chronic stress and depression are major contributors to FI, which can increase cortisol levels and disrupt the hypothalamic-pituitary-adrenal axis (HPA). A study by Prinz et al. [51] found that women, but not men, awoke earlier when their cortisol levels were higher. Insomnia and partial sleep deprivation are also linked to high cortisol levels [52]. In turn, sleep disturbances can alter appetite regulation by increasing ghrelin and decreasing leptin levels. In response to leptin, the hypothalamic receptors reduce appetite and increase energy expenditure. A low leptin level has also been associated with a higher body mass index, sleep disorders, and a greater likelihood of depressive symptoms. A low leptin level and a high ghrelin level are associated with sleep disturbances [53,54].

There are limited studies that investigate the link between food insecurity, sleep, and obesity. Across age groups, sexes, and races/ethnicities, Sawadogo's study [55] found a positive relationship between food insecurity and childhood obesity. According to their study, the association between food insecurity and sleep duration, as well as the association between sleep duration and obesity, were both stronger among males and non-Hispanic Asian children, resulting in a stronger proportion-mediated effect [55]. K. Do et al. examine associations between sleep, food insecurity, and weight status among adolescents. According to their research, a regular bedtime decreased the risk of obesity, difficulties staying asleep increased the risk of being underweight, and food insecurity increased the risk of being overweight [56]. In a study by Narcisse et al. [57], they examined the mediating role of sleep quality and quantity in the relationship between food insecurity and obesity. It was found that food insecurity and sleep quality were positively associated with obesity across racial/ethnic groups. There was also a direct correlation between food insecurity and sleep quantity in obese adults. The researchers propose that food insecurity may disrupt sleep and that perhaps through poor sleep, food insecurity may increase the risk of obesity [57]. The association between FI and obesity may be explained by some factors. In lower-income communities, food insecurity is more prevalent due to transportation limitations and a lack of access to fresh food and vegetables [58]. As a result of food insecurity, people tend to consume foods high in calories and fat, which increases their risk for obesity [59]. Furthermore, food insecurity contributes to irregular eating patterns characterized by periods of under consumption and food deprivation when resources are limited, and compensatory over consumption when resources are adequate, resulting in obesity [60]. Generally, food insecurity can disrupt sleep and increase obesity risk through inadequate sleep duration. The reason for the differences by age, sex, and race/ethnicity is not fully understood and future research should explore this.

There are several notable strengths to the current study. With a large sample size spanning six LMICs, our study is one of the few studies evaluating the associations between FI and multiple sleep outcomes among older adults in a multi-national context. Our study also has some limitations. The first limitation is that the data are cross-sectional, which made it difficult to infer causation. Second, although we controlled for several covariates in the analysis, other unmeasured factors such as noise and light level in participants' sleep environment could contribute to sleep quality and disturbances. Third, data were unavailable on the presence of sleep disorders such as insomnia and sleep apnea. Lastly, we assessed food insecurity and sleep outcomes based on self-reported questions, which can be subject to social desirability bias. Future research incorporating longitudinal follow-up are needed to confirm the observed associations and better understand the underlying mechanisms.

Our results contribute to the expanding body of knowledge on food insecurity and sleep related outcomes. These findings underline the necessity for further social services and public health initiatives that both address food insecurity and the coping mechanisms associated with it while improving sleep status among older adult in low‐ and middle‐income countries. For the purpose of developing beneficial programs or policies to assist individuals most impacted by food insecurity and poor sleep outcomes, it is essential to comprehend the relationships between food insecurity and sleep duration, quality, and disturbance.

## 5 Conclusion

In conclusion, the results of the current study from extensive data from six LMIC countries revealed that FI was associated with sleep duration, quality and disturbance among older adults.

## Authors’ contributions

LA and PA participated in the study’s design and provided a critical review and editing of the manuscript. PA, NS, MR and MG drafted the manuscript. MSY and FA conducted the statistical analysis and PA participated in the data interpretation. CL provided critical review and editing of the manuscript. All authors read and approved the final manuscript.

## Availability of data and materials

Datasets from Ghana, China, Russian Federation, India, South Africa and Mexico are available in Study on global AGEing and adult health (SAGE) website (https://apps.who.int/healthinfo/systems/surveydata/index.php/catalog/sage).

## Ethics approval and consent to participate

We used secondary analysis of datasets from Study on global AGEing and adult health (SAGE).

## Consent for publication

Not applicable.

## Competing interests

The authors declare that they have no competing interests.
